# A 100%-complete sequence reveals unusually simple genomic features in the hot-spring red alga *Cyanidioschyzon merolae*

**DOI:** 10.1186/1741-7007-5-28

**Published:** 2007-07-10

**Authors:** Hisayoshi Nozaki, Hiroyoshi Takano, Osami Misumi, Kimihiro Terasawa, Motomichi Matsuzaki, Shinichiro Maruyama, Keiji Nishida, Fumi Yagisawa, Yamato Yoshida, Takayuki Fujiwara, Susumu Takio, Katsunori Tamura, Sung Jin Chung, Soichi Nakamura, Haruko Kuroiwa, Kan Tanaka, Naoki Sato, Tsuneyoshi Kuroiwa

**Affiliations:** 1 Department of Biological Sciences, Graduate School of Science, the University of Tokyo, Tokyo, Japan; 2 Graduate School of Science and Technology, Kumamoto University, Japan; 3 Department of Life Science, College of Science, Rikkyo (St. Paul's) University, Tokyo, Japan; 4 Research Information Center for Extremophile, Rikkyo (St. Paul's) University, Tokyo, Japan; 5 Department of Life Sciences, Graduate School of Arts and Sciences, the University of Tokyo, Tokyo, Japan; 6 Institute of Molecular and Cellular Biosciences, the University of Tokyo, Tokyo, Japan; 7 Department of Integrated Biosciences, Graduate School of Frontier Sciences, the University of Tokyo, Chiba, Japan; 8 Center for Marine Environment Studies, Kumamoto University, Kumamoto, Japan; 9 Laboratory of Cell and Functional Biology, Faculty of Science, University of the Ryukyus, Okinawa, Japan; 10 Radiation Research Center for Bio-Technology, Advanced Radiation Technology Institute, Korea Atomic Energy Research Institute, Jeollabuk-do, Korea

## Abstract

**Background:**

All previously reported eukaryotic nuclear genome sequences have been incomplete, especially in highly repeated units and chromosomal ends. Because repetitive DNA is important for many aspects of biology, complete chromosomal structures are fundamental for understanding eukaryotic cells. Our earlier, nearly complete genome sequence of the hot-spring red alga *Cyanidioschyzon merolae *revealed several unique features, including just three ribosomal DNA copies, very few introns, and a small total number of genes. However, because the exact structures of certain functionally important repeated elements remained ambiguous, that sequence was not complete. Obviously, those ambiguities needed to be resolved before the unique features of the *C. merolae *genome could be summarized, and the ambiguities could only be resolved by completing the sequence. Therefore, we aimed to complete all previous gaps and sequence all remaining chromosomal ends, and now report the first nuclear-genome sequence for any eukaryote that is 100% complete.

**Results:**

Our present complete sequence consists of 16546747 nucleotides covering 100% of the 20 linear chromosomes from telomere to telomere, representing the simple and unique chromosomal structures of the eukaryotic cell. We have unambiguously established that the *C. merolae *genome contains the smallest known histone-gene cluster, a unique telomeric repeat for all chromosomal ends, and an extremely low number of transposons.

**Conclusion:**

By virtue of these attributes and others that we had discovered previously, *C. merolae *appears to have the simplest nuclear genome of the non-symbiotic eukaryotes. These unusually simple genomic features in the 100% complete genome sequence of *C. merolae *are extremely useful for further studies of eukaryotic cells.

## Background

The biological sciences have been embracing a new paradigm as a result of accruing genome information [1-13]. However, all previously reported eukaryotic nuclear genome sequences have been incomplete, especially in highly repeated units and chromosomal ends. Because repetitive DNA is essential to genome function [14], and may contribute to the diversity of isoforms [15] and the evolution of life [16], complete chromosomal structures are fundamental for understanding eukaryotic cells.

Our recently published nuclear-genome sequence of the ultra-small, hot-spring red alga, *Cyanidioschyzon merolae *10D, revealed some unique features, such as very few introns, only three copies of ribosomal (r)DNA, and a small total number of genes [9,10]. However, because uncertainties remained regarding the features of certain important repeated elements, such as histone-gene clusters and telomeres [9], this sequence, like all previous eukaryotic nuclear-genome sequences, was incomplete. Given the functional significance of such elements [14], it was obviously desirable to complete the sequence and resolve all ambiguities before attempting to summarize all of the unique features of the *C. merolae *genome. Therefore, we aimed to complete all previous gaps and sequenced all the remaining chromosomal ends, to construct the first nuclear genome sequence that is 100% complete. The results demonstrated that *C. merolae *possesses the simplest nuclear genome known among non-symbiotic eukaryotes.

## Results and Discussion

### A 100%-complete genome sequence

**Figure 1:**
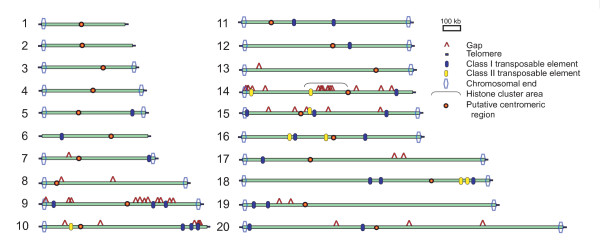
**Bird's-eye view of the 100% complete structures of 20 *C. merolae *chromosomes**. This shows regions that were filled in the present study ('Gap' and 'Chromosomal end'), telomere repeats, the histone cluster area, localization of transposable elements ('Class I' and 'Class II') and putative centromeric regions [9].

Completing the nuclear genome sequence, from telomere to telomere, without gaps or ambiguities, required two distinct subprojects: (i) filling in all 46 previously existing gaps between contigs, and (ii) sequencing the 34 (of 40) chromosome ends that had not been sequenced previously by the shotgun method [9]. Using PCR with sequence-specific primers to amplify portions of the *C. merolae *bacterial artificial chromosomes (BACs) that contained gap regions of interest, and then sequencing the resulting subclones, we have reduced the number of gaps between contigs from 46 to zero (Figure 1). A variety of methods (Additional files 1, 2) were then used to sequence all of the chromosomal ends that had not been sequenced previously (Figure 1). The global size of the 46 gaps that we have sequenced in this study is 46 469 base pairs. These sequences are G+C-rich (61%) compared with that of the whole nuclear genome (55%), and six of the 46 have extremely high G+C contents (70–76%). Furthermore, sequences corresponding to 13 previous gaps have extremely high nucleotide identity (99.4–100%) to other genomic regions. It turns out that the nuclear genomic sequence previously reported [9] contains misassembled and/or incorrect sequences of >20 kbp in total. The resulting complete genome sequence contains 16546747 base pairs and covers 100% of the chromosomal genome, from telomere to telomere. The numbers of base pairs in each of the 20 nuclear chromosomes are given in Table 1. These 20 linear nuclear chromosomes plus the two circular organellar DNA molecules [17,18] comprise the entire genome of the organism, and contain 16728945 base pairs (Table 1).

**Table 1:** Key features of the 22 chromosomes constituting the three genomes of the hot-spring red alga *Cyanidioschyzon merolae *10D

Genome/Chromosome	No. of nucleotides(bp)	Shape of chromosome	No. of protein-coding genes
Nucleus*			
1	422 616	Linear	102
2	457 013	Linear	125
3	481 791	Linear	144
4	513 455	Linear	140
5	528 682	Linear	161
6	536 163	Linear	131
7	584 452	Linear	173
8	739 753	Linear	213
9	810 151	Linear	231
10	839 707	Linear	247
11	852 849	Linear	236
12	859 119	Linear	258
13	866 983	Linear	249
14	852 727	Linear	256
15	902 900	Linear	265
16	908 485	Linear	261
17	1 232 258	Linear	355
18	1 253 087	Linear	360
19	1 282 939	Linear	384
20	1 621 617	Linear	484
Total	16 546 747		4 775
Unassigned	0		0
Plastid [17]	149 987	Circular	208
Mitochondrion [16]	32 211	Circular	34
Total of 3 genomes	16 728 945		5 017

*Based on the present 100% nuclear genome sequence.

### Histone-gene cluster

**Figure 2:**
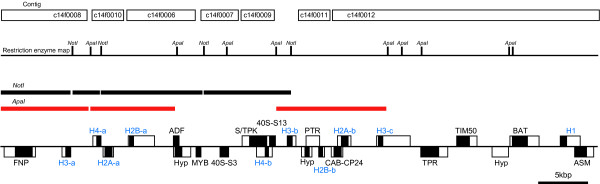
**Strategy for finishing the analysis of the *C. merolae *histone gene cluster on chromosome 14, and a map of histone genes resolved within the cluster**. The black and the white squares in genes represent CDS and 5'-/3'-untranslated regions, respectively. H4-a, histone H4-a; H2B-a, histone H2B-a; ADF, actin-depolymerizing factor; S/TPK, RIO-like serine/threonine protein kinase; 40S-S13, 40S ribosomal protein S13; H3-b, histone H3-b; PTR, possible transcribed region; H2A-b, histone H2A-b; H3-c, histone H3-c; TIM50, mitochondrial preseqence translocase subunit Tim50; BAT, brabched-chain-amino-acid transaminase, mitochondrial precursor; H1, histone H1; ASM, similar to N6-adenine-specific methylase; Hyp, hypothetical transcript; TPR, hypothetical protein, conserved (containing tetratricopeptide repeats); CAB-CP24, similar to chlorophyll a/b-binding protein, CP24; H2B-b, histone H2B-b; Hyp, hypothetical protein; H4-b, histone H4-b.

The total number of histone genes varies greatly by species. Animal genomes typically have several hundred to several thousand histone genes organized as a set of tandemly repeating quintets of the five major histone gene types [19,20]. In contrast, histone genes are located on more than one chromosome in some organisms, such as the ultra-small green alga *Ostreococcus *[12] and the flowering plant *Arabidopsis *[4]. In our previous study, the complete organization of the histone cluster area in the *C. merolae *genome was left unresolved, because of several gaps within this region of sequence [9]. However, our present complete sequence establishes that all *C. merolae *histone genes are localized on chromosome 14 and form a small cluster (around 48 kbp long) that includes two copies of the three core histone genes (*H2A*, *H2B *and *H4*), three copies of the *H3 *gene, and a single copy of the linker-histone *H1 *gene (Figure 2). In *Saccharomyces cerevisiae *[2], *Schizosaccharomyces pombe *[6], *Dictyostelium discoideum *[11], *Encephalitozoon cuniculi *[5] and *Ostreococcus tauri *[12] the histone genes also exhibit small copy numbers (up to three), as in *C. merolae*, but they are dispersed across more than two chromosomes. Thus, the *C. merolae *histone genes are present in the most compact cluster yet described.

### Telomeres and subtelomeric regions

Whole-genome shotgun sequencing suggested that the repeat unit in *C. merolae *telomeres is AATGGGGGG [9], but in that study only six of the 40 chromosome ends were examined [9] (Figure 1). Here the sequencing of all remaining termini confirmed that AATGGGGGG is the telomere repeat sequence in all *C. merolae *chromosomal ends. In most plants, the telomeres are composed of many copies of the sequence TTTAGGG [21], and the *C. merolae *telomere sequence, AATGGGGGG, has never yet been found elsewhere.

Telomere length varies among plants from approximately 0.5 kbp in the green alga *Chlorella vulgaris *to 150 kbp in tobacco [22,23]. Telomere restriction fragment analyses using the (CCCCCCATT)3 probe revealed AATGGGGGG repeats varying from 400 to 700 bp in the total chromosomal ends in the *C. merolae *genome (data not shown). Our Southern blot analysis using a specific genomic probe suggested that *C. merolae *chromosome 15 has around a 400-bp telomere repeat sequence at the left end (see Additional file 3). However, the longest telomeric repeats that could be sequenced in this study were 2.5 repeats (AATGGGGGGAATGGGGGGAATGGG) in the right end of chromosome 1, possibly because long stretches of AATGGGGGG repeats are difficult to clone or sequence using conventional techniques used in this and previous studies [9].

The putative telomerase catalytic subunit, telomerase reverse transcriptase (TERT) is transcribed (CMD110C) in *C. merolae *[9]. In the *C. merolae *genome, we found two possible telomerase RNA subunit genes in chromosomes 13 and 16, based on two transcripts, CMM123T and CMP131T, which included UUCCCCCCAUU and CCAUUCCCCCCAUU sequences, respectively. The telomerase RNA template sequence (CCCCCCAUU) has been detected in only these two hypothetical non-coding RNA genes among all the predicted genes in the present 100% complete nuclear genome. These are the first convincing candidate telomerase-RNA subunit genes in plant and algal lineages.

**Figure 3:**
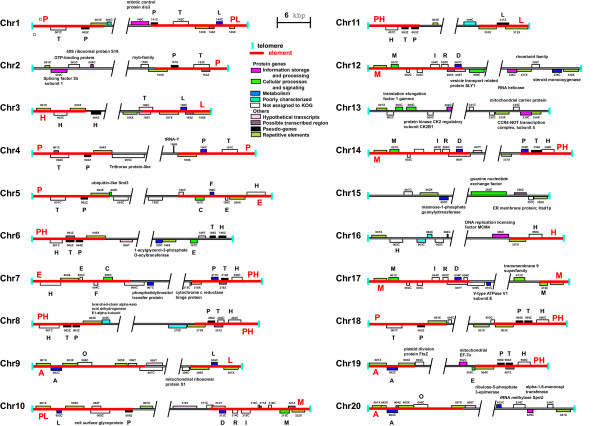
**Complete gene contents at the terminal regions of *C. merolae *chromosomes**. T, P, L, H, C, F, E, A, O, D, R, I and M represent genes for the trefoil factor, NADPH:protochlorophyllide oxidoreductase, L-lactate dehydrogenase, hedgehog protein, subunit 6a of chaperonin containing TCP1, subunit ATP5 of F_1_F_O _ATP synthase, ubiquitin conjugating enzyme E2, copper-containing amine oxidase, anion transporter, dTDP-glucose 4,6-dehydratase, dTDP-4-dehydrorhamnose reductase, iron permease, and dolichyl-phosphate-mannose:protein mannosyltransferase, respectively. Detailed information is available on the *C. merolae *Genome Project website . Homologous DNA elements are shown as red lines with designations.

Based on the end sequencing of all 40 chromosomal termini completed here, all of the subtelomeric regions in the *C. merolae *nuclear genome have been unveiled (Figure 3). Eight types of homologous DNA elements (up to 20 kbp), with unique gene contents, were found adjacent to the telomere repeats in the 40 chromosomal ends (Figure 3). Two of the eight types exist in only two ends; element H for 3L (left end of chromosome 3) and 16R, and element L for 3R and 11R. PH elements are the most frequent and are recognized in 6L, 7R, 8L, 8R, 11L, 14R, 18R and 19R, whereas nine termini (namely, 2L, 6R, 12R, 13L, 13R, 15L, 15R, 16L and 20R) showed no homology to other terminal regions. This situation is different from that of *S. cerevisiae*, in which core X elements exist in all chromosomal ends [24]. In the ultrasmall, obligate intracellular parasite *E. cuniculi *(Microsporidia) [5] and the nucleomorphs (the reduced nuclei of the eukaryotic endosymbionts) of the cryptophyte *Guillardia theta *and chlorarachniophyte *Bigelowiella natans *[25-27], both of the subtelomeric regions of all chromosomes harbor rRNA genes. However, such features are not recognized in *Cyanidioschyzon *(Figures 1, 3) and the ultra-small green *Ostreococcus *[12], and thus seem to represent some evolutionary convergence resulting from the intracellular parasitism or endosymbiosis in *E. cuniculi *and the nucleomorphs.

### Transposable elements

The number of transposable elements in various eukaryotes differs widely [28-30], and of all the non-symbiotic and non-pathogenic eukaryotes previously studied, yeast and pufferfish have the fewest: about 3% of the genome [29,30]. However, transposons constitute only about 0.7% of the *C. merolae *genome (by assuming that the average gene size of RT- and transposase-related elements are 4 kbp and 3 kbp, respectively). The completed *C. merolae *genome sequence contains only 26 class I elements (retrotransposons) and eight class II elements (transposons) [31] (Figure 1). The transcripts of three of the 26 retrotransposons contained an intact reverse transcriptase open-reading frame, and a BLASTX search suggested that all 26 of the retrotransposons were most closely related to non-long terminal repeat (non-LTR) retrotransposons. So, although LTR retrotransposons are widely distributed in plants and animals [31], we could not find any of them in the *C. merolae *genome. All eight of the transposase-related elements of *C. merolae *were most closely related (E-value = 0.071–6 × 10^-7^) to transposase sequences of bacterial transposons. In short, *C. merolae *is one of the two non-symbiotic eukaryotes with an extremely low abundance of transposable elements (Table 2). In the endosymbiotic reduced eukaryotes, *Encephalitozoon cuniculi *and nucleomorphs [5,25-27], however, transposable elements seem lacking in chromosomes.

**Table 2:** Comparison of the nuclear genomes of *Cyanidioschyzon*, *Ostreococcus *(an ultra-small green alga), *Arabidopsis *(a flowering plant) and *Ashbya *(a filamentous fungal pathogen).

**Organism**	**No. of protein-coding genes**	**Genes with introns (%)**	**No. of rRNA gene units**	**No. of chromosomes with histone genes**	**Transposable elements in genome (%)**	**Telomere repeat sequences**
*Arabidopsis*	26207	79	~800	5≤	~15	TTTAGGG
*Ostreococcus*	8166	39	4	6≤	~10	TTTAGGG
*Cyanidioschyzon*	4775	**0.5**	**3**	**1**	0.7	AATGGGGGG
*Ashbya*	**4718**	5	~50	4≤	**0.1>**	CGCTGAGAGACCCATACACCACAC

Bold type indicates the smallest number in non-symbiotic eukaryotes.

Two non-symbiotic eukaryotes, *Schizosaccharomyces pombe *and the filamentous fungus *Ashbya gossypii*, have nuclear protein-coding genes that are as small in number as those of *C. merolae*.

The number of protein-coding genes in the nuclear genome of *S. pombe *[6] increased to 5004 .

Although *A. gossypii *was reported to contain 4718 protein-coding nuclear genes [13], the genome project of this fungus is now in progress ; thus it possibly contains more than 4775 protein-coding genes.

In contrast to this, 253 copies of a novel interspersed repetitive element were found in the *C. merolae *genome. All copies have a truncated ORF that is weakly related (BLASTX E-value = 10^-5^-4 × 10^-2^) to a putative protein, WSV486, that is encoded in the genome of the shrimp spot syndrome virus [32]. These repetitive elements have an average size of 3.2 kbp, are distributed randomly on all chromosomes, and altogether comprise about 5% of the genome. Because these elements exhibit transcriptional activity [9], they may contribute to genomic or cellular functions in *C. merolae *in the same manner as repetitive DNA does in other eukaryotes [14].

## Conclusion

The smallest known histone-gene cluster, a unique telomeric repeat, a very low density of transposable elements, and other previously described simple features of the *C. merolae *nuclear genome [9,10] (Table 2) are very distinctive, and constitute the simplest set of genomic features found in any non-symbiotic eukaryote yet studied. Such simple features are generally considered to result from consequences of reductive evolution of an ultra-small eukaryote [12]. However, none of these features is shared by the similarly ultra-small green alga, *Ostreococcus*, in which 39% of the genes contain introns, histone genes are dispersed across at least six chromosomes, and 417 transposable elements and 8166 protein-coding genes are distributed among the chromosomes [12] (see Table 2). These may suggest differences in modes of genome reduction between ancestors of *Cyanidioschyzon *(red algae) and *Ostreococcus *(green plants). On the other hand, algae living in acidic hot springs (pH 1.5, 45°C) might be candidates for retaining ancient plant attributes, because the volcano activity is thought to have been providing such an extreme environment throughout Earth's history. Very recently, Cunningham *et al *[33] reported that *C. merolae *contains perhaps the simplest assortment of chlorophylls and carotenoids found in any eukaryotic photosynthetic organism. In addition, the *C. merolae *plastid genome contains a large number of genes, which is thought to be a primitive feature, because reversal of plastid-gene loss is generally considered to be impossible [34,35]. Thus, our hypothesis is that some of the unusual or simple genomic characteristics of *C. merolae *may represent primitive features that have been conserved in *Cyanidioschyzon*, but have become extensively modified during the evolution of other plant lineages. Alternatively, the unique genomic features of *C. merolae *(Table 2) may reflect adaptation to the extreme environment. However, genome information for other hot-spring red algae is very limited. The recently released nuclear genome sequence of another hot-spring red alga *Galdieria *has not revealed the chromosomal structures of its components, such as rDNA units or histone cluster area. [36]. Further information on the complete nuclear genomes of other plants, including other hot-spring red algae, red macro-algae, and other members of plant and algal lineages, will be needed to determine whether *C. merolae *actually has primitive genomic features.

Three kinds of genomes are found in many eukaryotic cells: nuclear, mitochondrial, and plastid [37]. Based on the present nuclear genome data and the previously published mitochondrial and plastid genome sequences [18,19], all major types of eukaryotic genetic information are present in *C. merolae*. In addition, as revealed by the present 100% complete genome, *C. merolae *contains unusually simple sets of genes and sequences (Table 2). For example, because almost all protein-coding nuclear genes of *C. merolae *lack introns (Table 2), the complete sequence of the genome provided here can be used directly to deduce the sequences of all of its proteins, which will make it extremely valuable for future proteomics research. Therefore, *C. merolae *represents an ideal model organism for studying the fundamental relationships among the chloroplast, mitochondrial and nuclear of genomes. The complete nuclear genome sequence reported here will greatly improve the precision of biological analyses of *C. merolae*, including studies of chromosome structure and gene structure/annotation. Furthermore, because *C. merolae *inhabits hot springs (45°C) [9], most of its proteins must be unusually heat-stable, and so its proteome may well provide important new insights into the structural basis for heat stability of proteins.

## Methods

### Filling gaps between contigs/fragments

Previously constructed *C. merolae *BAC clones [9] were used for filling in previously existing gaps between contigs. PCRs of BAC clones containing unsequenced regions were carried out using *Taq *polymerase with GC buffer (Takara LA Taq; Takara Bio Inc., Osaka, Japan) and specific primers complementary to sequences flanking the gaps. DNA walking annealing control primer technology (DNA Walking SpeedUp™ Kit; Seegene, Seoul, Korea) was also used to directly amplify unknown sequences adjacent to known sequences within a contig. PCR products were sequenced by cycle sequencing (Big-Dye Terminator Cycle Sequencing Kit v 3.1; Applied Biosystems, Foster City, CA, USA), except for a single gap in chromosome 10, which was filled by a sequencing reaction performed using *in vitro *transcription (CUGA Sequencing Kit; Nippon Genetech Co., Ltd., Tokyo, Japan).

### Sequencing of each chromosomal end

PolyC-tailing and the anchor primer method, the inverse PCR method and the asymmetric PCR method were used for sequencing the ends of chromosomes. For details, see Additional files 1 and 2.

### Assembling sequence data and gene annotation

Assembling of sequence data and two strategies for gene prediction have been described previously [9].

### Complete determination of the histone cluster area

To determine the complete sequences of the histone cluster area in the *C. merolae *genome, we carried out *Not*I and *Apa*I subcloning of the BAC clone GESZ2-b20, which included possible histone clusters in chromosome 14 [9]. Restriction-enzyme analysis, Southern blot analysis with histone-related probes and end sequencing of the subclones revealed relative positions of the subclones on chromosome 14 (Figure 2). Six gaps between contigs/fragments in the histone cluster area [9] were filled using primer walking of the subclones. For details, see Additional files 1 and 4.

### Accession numbers

The 100% chromosome sequences are accessible under the DDBJ with accession numbers AP006483–AP006502 (chromosome 1–20). Sequences and annotation are available at .

## Authors' contributions

HN, OM, KN, FY, YY, TF, SN, HK and TK performed filling contig gaps. HT, SM, KatT, SJC and KanT determined the chromosomal ends. KiT and NS completed the histone cluster area. MM and OM assembled the sequence data. ST analysed the transposable elements. All authors discussed the results and commented on the manuscript.

## Supplementary Material

Additional file 1 **Supplementary methods**: Cloning and sequencing of terminal regions of all chromosomes, and complete determination of the histone cluster area in *C. merolae*. Click here for file

Additional file 2 **Table 2**. Primers used for determination of terminal sequences of *C. merolae *chromosomes. Click here for file

Additional file 3 **Figure 5**. Telomere length analyses. (**A**) Southern hybridisation using the probe specific for the left arm of chromosome 15, demonstrating that the left end of chromosome 15 was detected using genomic DNA digested with each enzyme. H, Sa and Sp indicate genomic DNA digested with *Hin*dIII, *Sal*I and *Sph*I, respectively. (**B**) Comparison of the detected signal size with the fragment size estimated from the genome sequences. Click here for file

Additional file 4 **Table 3**. Primers used for completing the *C. merolae *histone cluster area. Click here for file
