# Supplementary material for: A 100%-complete sequence reveals unusually simple genomic features in the hot-spring red alga Cyanidioschyzon merolae
Source: BMC Biol. 2007 Jul 10;5:28. doi: 10.1186/1741-7007-5-28 (PMC1955436; doi:10.1186/1741-7007-5-28)
Supplement: Additional file 1 — Supplementary methods: Cloning and sequencing of terminal regions of all chromosomes, and complete determination of the histone cluster area in C. merolae. [file 1741-7007-5-28-S1.doc]

## Additional file 1

## Supplementary Methods

**Cloning and sequencing of terminal regions of all chromosomes of *C. merolae*.** For PCR amplification of chromosome ends, polyC-tailing and anchor primers were used according to the methods of Lopez et al. [S1]. Template DNA was prepared from appropriate chromosome bands after *C. merolae* cells were subjected to pulsed-field gel electrophoresis (PFGE). When we initially attempted to amplify the right terminus of chromosome 1, 50 ng DNA including chromosomes 1 to 5 were used.

Chromosome DNA was suspended in the tailing buffer containing 0.75 mM cobalt chloride, 750 µM dCTP and 37.5 µM ddCTP, incubated at 50°C for 10 min and cooled down to 37°C. Twenty units of terminal transferase (F. Hoffmann-La Roche Ltd., Basel, Switzerland) were then added, and the mixture was incubated at 37°C for 90 min. The enzyme was inactivated at 75°C for 10 min. After ethanol precipitation, a tailed DNA sample was resuspended with 1x Ex Taq buffer (Takara Bio Inc., Osaka, Japan) with 0.2 mM of each dNTP, and half of the mixture was used for asymmetric PCR with 0.1 µM of the anchor primer R_0_-R_1_-dG_14_ (5’-GCTAAGTCGGGAGCTCTACAAGC-AAGGATCCGTCGACATC-G_14_-3’) [S1]. With 2.5 units of Takara Ex Taq polymerase (Takara Bio Inc.), ten cycles of asymmetric PCR were run at 94°C for 1 min, 59°C for 2 min and 72°C for 2 min. The amplified DNA was then isolated with a PCR purification kit (Qiagen, Hilden, Germany) according to the manufacturer’s instructions. The 1/10 volume of purified DNA sample served as a template for a second PCR step with the primer R_0_, which fit the R_0_-R_1_-dG_14_ sequence, and a terminal region-specific primer (5’-ACCCTCTTCGCTAGGCAATATG-3’) for the right end of chromosome 1. The reaction mixture contained DNA sample, 0.2 mM dNTP, 0.2 µM of each primer and 2.5U of Ex Taq polymerase in 1x reaction buffer supplemented with polymerase. We used a touchdown PCR method. After 2 min of incubation at 94°C, the reaction consisted of seven cycles with a 1-min denaturation at 95°C, a 1-min annealing starting at 65°C and decreasing by 1°C during each of the subsequent seven cycles, and a 3-min elongation at 72°C. For the following 28 cycles, the annealing temperature was held at 58°C. The last cycle was terminated with a final 7-min extension at 72°C. The PCR products were subject to agarose gel electrophoresis, and amplified DNAs >1.5 kbp were purified from the gel. Isolated DNAs were used for nested PCR with the primer R_1_ nested terminal region-specific primer (5’-AACGGGTGCTCAAGGTAGGTA-3’) and Ex Taq polymerase with a 2-min denaturation at 94°C and 32 cycles at 94°C for 30 sec, 55°C for 30 sec and 72°C for 1 min with a final 10-min extension at 72°C. The nested PCR sample was subject to agarose gel electrophoresis. Amplified DNA in the band was purified, cloned into the pT7Blue vector (Novagen, San Diego, CA, USA) and sequenced with a CEQ2000 sequencer (Beckman Coulter, Inc., CA, USA), a Model 3100 sequencer (Applied Biosystems, Foster City, CA, USA) or IR4200 (LI-COR, Lincoln, NE, USA).

We employed an inverse PCR method to determine the terminal sequence of the left end of chromosome 4. Briefly, total genomic DNA was digested by either *Bam*HI or *Eco*RV, blunted by T4 DNA polymerase (Takara Bio Inc.) and circularised by T4 DNA ligase (New England Biolabs, Beverly, MA, USA). The resultant circular self-ligates were purified by phenol/chloroform extraction and used as templates for the subsequent nested PCRs. First and second PCR products were amplified with LA Taq polymerase using nested primer sets (first primer set; 5’-ACGCCTCCGAGCACCAACAT-3’ and 5’-CCACTGCCGCATATCGAACC-3’: second primer set; 5’-GGCGAGTTCTTAGTACGGTG-3’ and 5’-GCCTAGCGAAATCAATCCCA-3’) with a 2.5-min denaturation at 96°C and 30 cycles at 96°C for 30 s, 60°C for 30 s and 72°C for 3 min. Fragments containing the terminal region were excised from the agarose gel, cloned into pGEM-T Easy vector (Promega, Madison, WI, USA) and sequenced.

To determine telomere sequences of all chromosomal ends, an asymmetric PCR method was used with the telomere-specific primer, which contains telomere repeats, and R_0_-R_1_ sequences (5’-GCTAAGTCGGGAGCTCTACAAGCAAGGATCCGTCGACATC ATTCCCCCCATT-3’; R_0_-R_1_ sequence is underlined). For the determination of terminal sequences of all chromosomal ends, the PFGE-isolated chromosomes used were chromosome 1 for the end of chromosome 1, chromosomes 1–5 for the ends of chromosomes 2 and 5, chromosome 7 for the end of chromosome 7, chromosome 8 for the end of chromosome 8, chromosomes 9–14 for the ends of chromosomes 9–14, chromosomes 9–16 for the ends of chromosomes 15 and16 and chromosomes 17–20 for the ends of chromosomes 17–20. Asymmetric PCR was performed with PFGE-isolated chromosomal DNA (approximately 10 ng), 0.2 mM dNTP, the telomere-specific primer (0.1 µM) and Ex or LA Taq polymerase in 1x attached buffer, with 2 min of denaturation at 94°C and ten cycles at 94°C for 1 min, 58°C for 2 min and 72°C for 2 min. The second PCR was carried out with the R_0_ or R_1_ primer, the specific primer for each chromosome end and Ex or LA Taq polymerase. Amplified samples were subjected to agarose gel electrophoresis, and amplified DNA in the appropriate band was purified, cloned into the pT7Blue vector and sequenced with a CEQ2000 sequencer. The specific primers used are summarized in Additional file 2.

## An alternative PCR method was used to determine the sequences of both ends of chromosome 3 and the right end of chromosome 4. The terminal regions of the chromosomes were amplified from the genomic DNA of *C. merolae* with LA Taq polymerase using the M13R-conjugated telomere-specific primer (5’-CAGGAAACAGCTATGACCCCCCCATT-3’; M13R primer sequence is underlined) and a chromosome-specific primer (Additional file 2) with one cycle at 96°C for 2 min, 50°C for 30 s and 72°C for 3 min. An aliquot of the PCR product was used as a template for the second PCR using M13R primer (5’-CAGGAAACAGCTATGA-3’) and the same chromosome-specific primer as the one used in the first PCR with a 2.5-min denaturation at 96°C and 30 cycles at 96°C for 30 s, 60°C for 30 s and 72°C for 3 min. Amplified fragments were cloned into pGEM-T Easy vector and sequenced. The sequence of the left end of chromosome 4 was confirmed by this alternative PCR method.

To determine the *C. merolae* telomere length *in vivo*, we conducted genomic Southern hybridisation using the probe specific to the left end of chromosome 15. The measurement of the band size indicated that signal positions corresponding to the chromosomal ends digested with several restriction enzymes (with no effects on telomere repeats) were about 400 bp larger than the estimated length from restriction sites to telomere repeats in the *C. merolae* genome sequence (Additional file 3). Genomic Southern hybridisation analyses using a DIG labelling system (F. Hoffmann-La Roche Ltd.) were conducted following the manufacturer’s instructions. The probe specific for the left arm of chromosome 15 was generated from *C. merolae* genome DNA with 15L_F and 15L_R primers (15L_F, 5’-CGAGGACAAACACAGCAGAA-3’; 15L_R, 5’-ACTCTCCCTGGAGACAAGCA-3’). Band sizes were measured with an LAS-3000 image analyser (Fujifilm, Minamiashigara, Kanagawa, Japan).

### Complete determination of the histone cluster area. The construction of the pBS bridge plasmid was completed by subcloning techniques. BAC clone GESZ2-b20 (5 µg) was digested with *Not*I or *Apa*I at 37˚C overnight. The digestion products were cloned into the pBSII SK+ vector (Stratagene, La Jolla, CA, USA). These subclones were purified with Qiagen plasmid miniprep (Qiagen). The end sequence was determined using the plasmid as a template, with the BcaBEST Sequencing primer M13-20 and BcaBEST Sequencing primer RV-P (Additional file 4). To determine the position of the subclone in the chromosome, the restriction enzyme was processed with *Hin*CII, *Pst*I and *Hin*CII+*Pst*I. In addition, Southern blot analysis was conducted with the histone H2A, H2B, H3 and H4 specific digoxigenin labelling probes (PCR products using primers in Additional file 4), putative protein and a Y105E8B.3 specific fluorescein labelling probe. The *Not*I or *Apa*I subclone plasmid as a template and the primer walking methodology were used for sequencing the contig gap regions.

## Reference

S1. Lopez CC, Neilsen L, Edström JE: Terminal long tandem repeats in

chromosomes from *Chironomus pallidivittatus*. 1996, *Mol Cell Biol* **16:** 3285–3290.
