# Supplementary material for: A 100%-complete sequence reveals unusually simple genomic features in the hot-spring red alga Cyanidioschyzon merolae
Source: BMC Biol. 2007 Jul 10;5:28. doi: 10.1186/1741-7007-5-28 (PMC1955436; doi:10.1186/1741-7007-5-28)
Supplement: Additional file 2 — Table 2. Primers used for determination of terminal sequences of C. merolae chromosomes. [file 1741-7007-5-28-S2.doc]

## Additional file 2

**Table 2.** Primers Used for Determination of Terminal Sequences of *C. merolae* Chromosomes.

| Chromosome | Primer name | Sequences (from 5’ to 3’) |
| --- | --- | --- |
| 1L* | 839-3 | TCGTTGACAAAGAGAGGCCAGG |
| 2R* | 898-2 | CCAAAGCGTGCTTGGTGCAAG |
| 3L | c756h_R(1074) | TCGGCACTAAGTCACGTTTC |
| 3R | c927tF321711 | GACCATACAGTTGCGAGTGA |
| 4L | c758head_R | GAAGGAAGACTCAGCCAAAC |
| 4R | c926t_Inv_F | GGGTCGCATCCAAACAAAAG |
| 5L | 724-1 | CGATCGCAGTCGATACAATGC |
| 5R | 680-2 | TGGGCGTTATGTACAGGTTCTCC |
| 7L | 680-2 | TGGGCGTTATGTACAGGTTCTCC |
| 7R | 878-4 | AAGCAGCAGCAGCAGCATCAGAAGC |
| 8L | 875-2 | CAGGGTGACAAAAGAGTCTTGA |
| 8R | 866-2 | GATGACTACTGTGGATATTCGGA |
| 9L | 721-2 | CTCTGGAATTAGCTACTATGTCAC |
| 9R | CmT3 | ACCCTCTTCGCTAGGCAATATG |
| 10L | CmT2 | AACGGGTGCTCAAGGTAGGTA |
| 11L | 866-4 | AATAGAAATAAAGTGCCCACGGTCA |
| 11R | 880-2 | ACACCCTAGCATCCCATCCGAA |
| 12L | 619-1 | AATGCCGAGCGCATAGCCAAGC |
| 12R | 917-2 | ACACGGCAGGAAGCGGAATACA |
| 13L | 798-2 | AAAAAGGATAATCATGACGGACACA |
| 13R | 869-1 | GTAGTCATTCTCGCGATTATGT |
| 14L | 619-1 | AATGCCGAGCGCATAGCCAAGC |
| 15L | 784-2 | TTTGCACGATCCGGGACTACAA |
| 15R | 907-1 | TAATCGCGTGTACTCCGTGTGAC |
| 16L | 862-2 | CGAACGCATTTGATTGTTGGAT |
| 16R | 882-2 | TTTTGGACCGGACACCTATTAC |
| 17L | 637-2 | ATTGTCCTTGAACCTATGTTGC |
| 17R | 619-2 | ACCGCTGCCAAGCACATCCAGA |
| 18L | 898-2 | CCAAAGCGTGCTTGGTGCAAG |
| 18R | 866-2 | GATGACTACTGTGGATATTCGGA |
| 19L | 721-2 | CTCTGGAATTAGCTACTATGTCAC |
| 19R | 866-2 | GATGACTACTGTGGATATTCGGA |
| 20L | 721-2 | CTCTGGAATTAGCTACTATGTCAC |
| 20R | 915-1 | TCAGAATCTCACTTTGGGCAAGG |

*Symbols L and R at the chromosome number show left and right termini, respectively.
