# Supplementary material for: A 100%-complete sequence reveals unusually simple genomic features in the hot-spring red alga Cyanidioschyzon merolae
Source: BMC Biol. 2007 Jul 10;5:28. doi: 10.1186/1741-7007-5-28 (PMC1955436; doi:10.1186/1741-7007-5-28)
Supplement: Additional file 3 — Figure 5. Telomere length analyses. (A) Southern hybridisation using the probe specific for the left arm of chromosome 15, demonstrating that the left end of chromosome 15 was detected using genomic DNA digested with each enzyme. H, Sa and Sp indicate genomic DNA digested with HindIII, SalI and SphI, respectively. (B) Comparison of the detected signal size with the fragment size estimated from the genome sequences. [file 1741-7007-5-28-S3.doc]

## Additional file 3


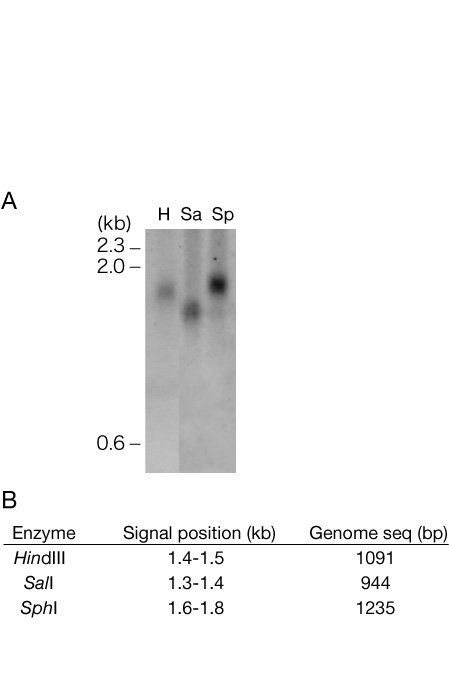


**Figure 5.** Telomere Length Analyses.

1. Southern hybridisation using the probe specific for the left arm of chromosome 15, demonstrating that the left end of chromosome 15 was detected using genomic DNA digested with each enzyme. H, Sa and Sp indicate genomic DNA digested with *Hin*dIII, *Sal*I and *Sph*I, respectively.
2. Comparison of the detected signal size with the fragment size estimated from the genome sequences.
