# Supplementary material for: A 100%-complete sequence reveals unusually simple genomic features in the hot-spring red alga Cyanidioschyzon merolae
Source: BMC Biol. 2007 Jul 10;5:28. doi: 10.1186/1741-7007-5-28 (PMC1955436; doi:10.1186/1741-7007-5-28)
Supplement: Additional file 4 — Table 3. Primers used for completing the C. merolae histone cluster area. [file 1741-7007-5-28-S4.doc]

**Additional file 4**

**Table 3.** Primers Used for Completing the *C. merolae* Histone Cluster Area.

For sequencing

BcaBEST Seqencing primer M13-20 CGACGTTGTAAAACGACGGCCAGT

BcaBEST Seqencing primer RV-P GGAAACAGCTATGACCATGATTAC

For Southern blotting

H3

786-1450F CCGACTACATCTGCCACGACGATTCCGTTT

786-1966R ATATCCTTCGGCATAATCGTCACACGCTTC

H2A

867-87715F ATGATCGACACCTGCGCATCGCGCGCAGCC

867-87097R TTTCTTCTTCGGGAGAAGCACTGCGTGGAT

H2B

607-192F ATGGCTATGAAAGGGAAGACTGTTGCGAAG

607-546R CTTGAGGTGTATTTGGTGACAGCCTTGGTG

putative

607-1289F ATGGCAGCTTGTCATTTCTCATAAATGTCC

607-1628R CAAAGCGCCCTCTGTCGGAATGGCGGAACG

H4

616-409F ATGTCAGGTCGTGGCAAGGGCGGCAAGGGT

616-1114R TACAGAGGCTGACGTTGGCGTTGCGAGTGC

Y105E8.3

616-2436F TCGCACTTTAACGGCCGTGGAGCTGGGTAT

616-976R CAAGTCGCGTTGCTTCGCGGAGCAACAGTT
